# The Beat

**Published:** 2009-06

**Authors:** Erin E. Dooley

## More Weight, More Allergies?

A study co-funded by the NIEHS and the NIAID suggests a possible connection between rising rates of childhood obesity and allergies over the past few decades. Analyzing 2005–2006 NHANES data, the researchers reported in the May 2009 *Journal of Allergy and Clinical Immunology* that obese children were 26% more likely than children of normal weight to have allergies and 59% more likely to have a food allergy. Although an increased risk of allergy might not be the most serious health problem faced by overweight children, it does provide an added incentive for stepping up efforts to prevent childhood obesity, the researchers contend.

**Figure:**
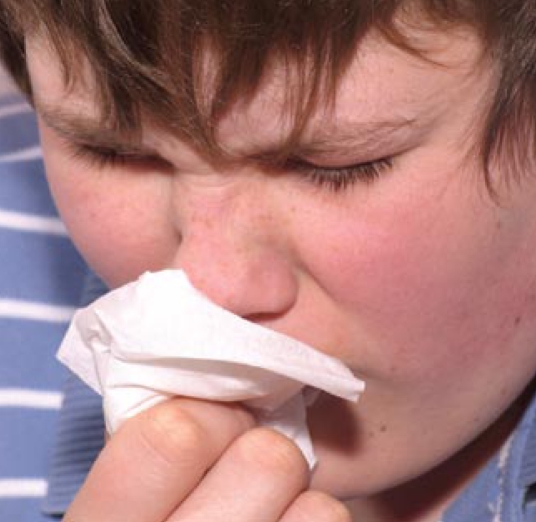


## Some “Lead-Free” Pots Are Not

Traditional lead-glazed pottery from Mexico has been cited as a common source of lead exposure among people who use these wares. A study by the Environmental Quality Institute at the University of North Carolina at Asheville showed that Mexican-made “Mi Pueblo” brand clay pots marketed in some eastern U.S. states as “lead-free” actually contained nearly twice the amount of lead considered safe by the FDA for cups, mugs, and pitchers, and just under the safe level for serving bowls. Ironically, the researchers had hoped to confirm the safety of Mi Pueblo products to enable their promotion as a safe alternative to lead-glazed pots.

**Figure:**
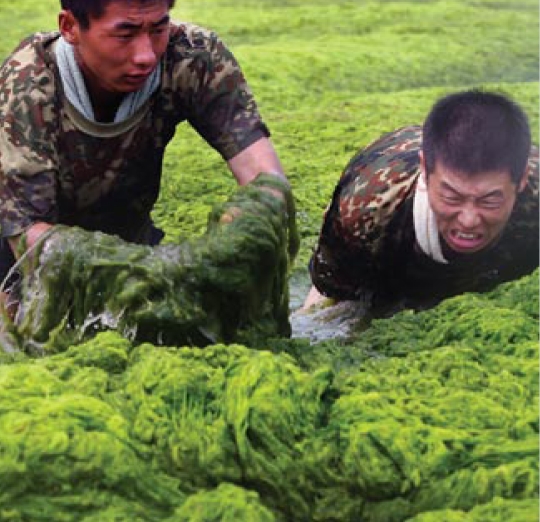
Military officers clean up the Qingdao algal bloom, July 2008

## Massive Bloom the New Norm?

In summer 2008 an unprecedented algal bloom almost caused the cancellation of the Olympic Games’ sailing events in the regatta city of Qingdao, China. It took more than 10,000 people to clean up over 1 million tons of *Enteromorpha prolifera* algae from the beach and coast. Initially attributed to eutrophication, a study in the June 2009 *Marine Pollution Bulletin* now suggests a different provenance for the bloom. The authors used satellite images to trace the bloom’s origin to the coast of Jiangsu province, where *E. prolifera* contaminates aquaculture operations that grow *Porphyra* seaweed for food. With the aquaculture industry off Jiangsu’s coast having more than doubled in size since 2003, the authors say the stage is set for more massive blooms such as the one seen in Qingdao.

## Carbofuran Banned for Food Crops

On 11 May 2009 the EPA revoked regulations that allowed small residues of the pesticide carbofuran on food crops, saying the compound poses an unacceptable health risk, especially to children and farmworkers. The move came after years of review, a partial ban in the mid-1990s (when millions of migratory bird deaths were linked to carbofuran), and an attempt earlier in 2009 by the chemical’s manufacturers to delay further restrictions. The EPA cited unacceptable neurotoxicity and other health risks posed by ingesting residues of the insecticide in food and water. The final carbofuran tolerance rule takes effect in December 2009.

## BPA Becoming Chemical *Non Grata*

In May 2009 Chicago and Minnesota became the first city and state, respectively, to ban bisphenol A (BPA) in baby bottles and children’s cups. Lawmakers in Connecticut and California are now considering similar BPA regulations. On the federal level, bills aimed at banning BPA from all food and beverage containers were introduced in the House and the Senate in March 2009 but are still in committee. BPA is found in some polycarbonate plastic containers such as water and baby bottles and in some food can linings. BPA is weakly estrogenic, and numerous animal studies suggest exposure to the compound during critical developmental windows may contribute to adverse reproductive, behavioral, and metabolic effects.

**Figure:**
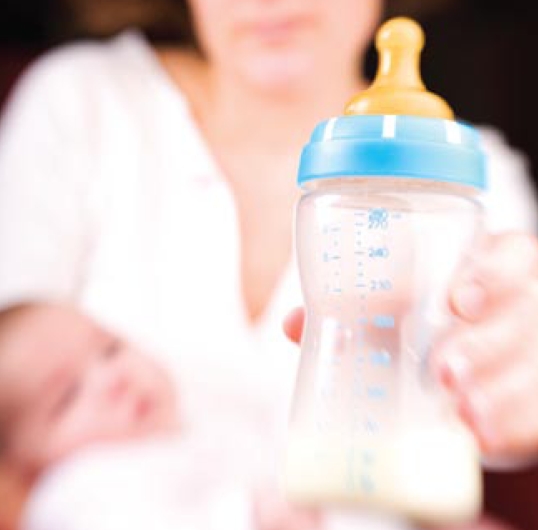


## Bad Air Rising

Six of 10 U.S. citizens, or more than 186 million people, reside in areas with dangerous levels of air pollution, according to the American Lung Association’s *State of the Air: 2009* report. Although some major U.S. cities have improved their air quality over the last decade, the air in many other cities became more polluted last year. The 2009 figures are substantially higher than those from 2008, when an estimated 42% of the population faced similar conditions. The report includes a call for the EPA to strengthen its standards for ozone and particulate matter. The agency issued new ozone standards in 2008, and proposed revisions to particulate matter standards are due in 2010.

